# Productiveness and Berry Quality of New Wine Grape Genotypes Grown under Drought Conditions in a Semi-Arid Wine-Producing Mediterranean Region

**DOI:** 10.3390/plants11101363

**Published:** 2022-05-20

**Authors:** Diego José Fernández-López, José Ignacio Fernández-Fernández, Celia Martínez-Mora, Juan Antonio Bleda-Sánchez, Leonor Ruiz-García

**Affiliations:** 1 Equipo de Mejora Genética Molecular, Departamento de Biotecnología, Genómica y Mejora Vegetal, Instituto Murciano de Investigación y Desarrollo Agrario y Medioambiental, 30150 Murcia, Spain; diegoj.fernandez@carm.es (D.J.F.-L.); celia.martinez@carm.es (C.M.-M.); 2 Equipo de Enología y Viticultura, Departamento de Desarrollo Rural, Enología y Agricultura Sostenible, Instituto Murciano de Investigación y Desarrollo Agrario y Medioambiental, 30150 Murcia, Spain; josei.fernandez@carm.es (J.I.F.-F.); juanantonio.bleda@carm.es (J.A.B.-S.)

**Keywords:** deficit irrigation, drought, grape quality, phenology, productivity

## Abstract

One alternative for adapting viticulture to high temperatures and the scarcity of water is the development of new varieties adapted to such conditions. This work describes six new genotypes, derived from “Monastrell” × “Cabernet Sauvignon” (MC16, MC19, MC72, MC80) and “Monastrell” × “Syrah” (MS104, MS49) crosses, grown under deficit irrigation and rainfed conditions in a semi-arid wine-producing area (Murcia, southeastern Spain). The effect of genotype, year, and irrigation treatment on the phenological, productiveness, morphological, and grape quality data was evaluated. The study material was obtained and selected as part of a breeding program run by the *Instituto Murciano de Investigación y Desarollo Agrario y Medioambiental* (IMIDA). The results obtained show that under rainfed conditions, the values for productive variables decreased, while those referring to the phenolic content increased. Notable variation in the parameters evaluated was also seen for the different genotypes studied. The behavior of the genotypes MC80 and MS104 under rainfed conditions was noteworthy. In addition to maintaining very adequate yields, phenolic contents, must pH, and total acidity values, MC80 fell into the best ‘phenolic quality group’ and MS104 returned a low º°Baumé value, ideal for the production of low-alcohol-content wines. These genotypes could favor the development of sustainable quality viticulture in dry and hot areas.

## 1. Introduction

One of the most severe abiotic stresses expected with climate change in the Mediterranean Basin is drought, which will doubtlessly be aggravated by increased temperatures and solar radiation [1]. The IPCC has reported that areas with Mediterranean climates are likely to face increased drought and reduced renewable surface water and groundwater resources [2]. Despite its ability to adapt to different environmental conditions, the grapevine is one of the most sensitive fruit crops with respect to water scarcity and severe drought; hence, it represents a major concern among viticulturists, winemakers, and enologists regarding the effects of climate change on the production and conservation of wine. This is especially true in the Mediterranean Basin where water resources are particularly vulnerable, and where most grapevine-growing areas are located [3,4,5]. The climatic scenario for the region, which involves increased drought and raised temperatures, will have consequences for vine development, such as the earlier appearance of the different phenological stages; indeed, this is already taking place [6,7]. Changes may also occur at the physiological level, and the qualitative characteristics of the grapes and eventual wine will likely be affected [8,9,10,11,12,13,14,15]. Smaller yields can be expected in line with reductions in berry and bunch weight, together with restricted growth, smaller leaf surface areas (with early senescence and premature leaf fall), increased respiration and evapotranspiration, and reduced photosynthetic activity [3,13,14,15,16,17].

Preventive and adaptive measures need to be taken by the wine sector if the adverse effects of climate change are to be mitigated [18,19,20,21]. A short-term preventive measure could be the use of deficit irrigation techniques, which may improve the quality of grapes and wine [22,23,24,25,26,27,28,29] while maintaining good yields—as long as the water-stress threshold is not exceeded (which would lead to a reduction in wine quality) [30]. However, Fraga et al. (2018) [31] observed that, in hot and dry regions in Portugal, yields were significantly reduced even when efficient irrigation was available, a consequence of water and heat stress; daytime temperatures above 35 °C negatively affect flowering and fruit set [32] and therefore yield. High temperatures have been correlated to the elevated synthesis of anthocyanins, although at temperatures above 35 °C, anthocyanins stop accumulating and may even be degraded [33,34,35]. Thus, in hot and dry regions, viticulture cannot be sustained simply by the use of deficit irrigation techniques; it will be necessary to adopt other measures to maintain the sustainability of the system. The selection of suitable plant material (variety/clone and rootstock) from the existing vine biodiversity is one of the most powerful long-term strategies for adapting wine production to water scarcity [1,3,13,36,37,38,39,40]. Another alternative is the development and selection, through directed crosses, of new vines that are better adapted to the specific conditions of the viticulture zone [37,41,42] while still showing good agronomic properties, grape quality, and enological characteristics [43,44]. Changes in vineyard management will, of course be needed too, combining efficient irrigation (if possible) with the use of more drought-tolerant plant material [19,31,45].

Different varieties adapted to current drought conditions have been identified, such as “Monastrell”, “Cabernet Sauvignon”, and “Syrah”, among others [9,22,30,46]. However, the quality of these varieties might fall with the higher temperatures and greater water scarcity expected in the coming years. In fact, climate change forecasts in semi-arid areas, where the availability of water is already limited, indicate that the climate will become warmer and drier, threatening the sustainability of vineyards in the near future [43,47]. Hence, the importance of taking adaptation measures in these areas, especially those related to obtaining and selecting new material that is better adapted to water scarcity and rising temperatures. Since the combination of temperatures > 35 °C and water scarcity can reduce yields and the concentration of polyphenols and anthocyanins in the berries, the selection of new varieties with higher-than-normal concentrations of polyphenols and anthocyanins is of great interest. Excessive sugar accumulation may also occur under hot conditions, with a consequent increase in the alcohol content of the eventual wine. Thus, varieties are also needed that ripen with a lower sugar concentration under such conditions. This would be of great interest since it might lead to products suitable for consumers who demand quality wines with lower alcohol contents.

The Instituto Murciano de Investigación y Desarollo Agrario y Medioambiental (IMIDA), in Murcia, Spain, has been running a program to develop grapevine varieties with better phenolic quality for semi-arid wine-producing areas since the 1990s. The program is based on new genotypes obtained from crosses between ‘Monastrell’ and other varieties such as Cabernet Sauvignon, Syrah, Tempranillo, Verdejo and Barbera [48,49,50]. ‘Monastrell’ is cultivated in different parts of Spain (particularly the southeast); it is the main variety grown in the Jumilla, Bullas and Yecla Denominations of Origin (occupying 81% of the cultivated area)—all of which have a semi-arid Mediterranean climate, with hot summers, mild winters, and scant rainfall that averages between 300 and 350 mm/year. It is also cultivated in France (where it is known as ‘Mourvedre’), California (where it is known as ‘Mataró’), and Chile, and in recent years, it has been increasingly planted in Australia [51].

Using study material produced within the above IMIDA breeding program, the present work examines the effect of genotype, year, and irrigation treatment (collecting phenological, productiveness, morphological, and grape quality data) for six new genotypes obtained via “Monastrell” × “Cabernet Sauvignon” and “Monastrell” × “Syrah” crosses, when grown under controlled deficit irrigation and rainfed conditions. The final objective of this work is the identification and selection of new genotypes best adapted to the conditions of drought and high temperatures in semi-arid zones, as a measure of adaptation to the adverse effects of climate change.

## 2. Results

### 2.1. Phenological Stages

Table 1 shows that for most of the phenological stages studied, significant variation (*p* < 0.001) existed among the genotypes and the year of study within the same irrigation treatment. No significant differences were found between irrigation treatments for the phenological stages.

The mean duration of the period from budbreak to total leaf fall was very similar under both the RDI and rainfed conditions (223 and 222 days, respectively). MS104 had the shortest mean duration of this phenological period under both (RDI 207 days, and rainfed 205 days), while MC72 had the longest (238 days and 237 days, respectively).

The ripening period, i.e., from the date of veraison to the date of harvest, averaged 29 days under RDI, and 28 days under the rainfed conditions. MC72 had the shortest maturation period under both conditions (mean 25 and 23 days, respectively), while MC16 had the longest under the RDI conditions (mean 36 days), and MC80 the longest under the rainfed conditions (mean 34 days). Taking into account the mean harvest date, MC16, MC80, and MS49 were the latest maturing genotypes, while MC19, MC72, and MS104 were the earliest (Table 1). Finally, the overall mean period of leaf fall, calculated from the starting date to total leaf fall, was 36 and 37 days under the RDI and rainfed conditions, respectively. Again, there were differences among the genotypes: MS104 had the longest period of leaf fall (mean 52 and 54 days under the RDI and rainfed conditions, respectively), whereas MC80 had the shortest (mean 24 and 25 days, respectively). MS104 entered its rest period the earliest (9–8 December), and MC72 the latest (5–6 December).

### 2.2. Yield Parameters

The yield values varied significantly among the genotypes (G), irrigation treatments (T), and year of study (Y) (Table 2), with the interaction G × Y strongest (*p* < 0.001) with respect to most yield variables. The interaction G × T × Y was significant (*p* < 0.001) only for the parameters related to the weight of the berry. The total yield (kg vine^−1^) was significantly lower under the rainfed conditions than under RDI, with a mean reduction for the study period (2018–2021) of 39.4%, mainly due to the reduced mean weight of the bunches (33.5%), berries (20.5%) and number of bunches (14.3%). MS104 was the most productive genotype under both the RDI (mean 2.52 kg vine^−1^) and rainfed (mean 1.51 kg vine^−1^) conditions, mainly due to a higher mean bunch weight under both (mean RDI 131.04 g, mean rainfed 87.46 g). MC19 and MC72 were the least productive under both RDI (mean 1.76 kg vine^−1^) and rainfed (mean value of 1.03 kg vine^−1^) conditions, probably because MC19 had one of the lowest number of bunches and MC72 one of the lowest mean bunch weights (Table 2).

MC80 returned the least affected total yield under the rainfed conditions compared to RDI, with a mean reduction of 21%, mainly due to less reduced bunch (18%) and berry weights (6%). MS49 was the genotype most affected by the rainfed conditions, with an average reduction of 54% compared to under RDI, mainly due to a greater reduction in the bunch (53%) and berry weights (41%). A progressive reduction in the mean total yield (i.e., of all genotypes) was also observed from 2018 to 2020, under both the RDI (48%) and rainfed conditions (62%), coinciding with a reduction in bunch weight (42% under RDI and 51% under rainfed conditions) and in the number of bunches (16% and 34%, respectively). Compared to 2020, in 2021, there was a recovery in the total yield (80% under RDI and 96% under rainfed conditions) and in the mean bunch weight (79% under RDI and 88% under rainfed conditions) (Table 2).

The berry weight variables were those most significantly influenced by the interactions G × T, G × Y and G × T × Y (*p* < 0.001). The mean berry weight was significantly reduced under the rainfed conditions compared to RDI, with a mean reduction of 20%; MS49 showed the mean berry weight most affected, with a reduction of 41% compared to 6% for MC80 (Table 2). The mean percentage contribution of the skin (%skin) and of the seeds (%seeds) to berry weight increased under the rainfed conditions by 5% and 23%, respectively, compared to RDI. MC80 was the genotype with the most increased %skin under the rainfed compared to the RDI conditions (57%). %skin values are a sign of quality; MC16 and MC80 returned the highest %skin contributions to berry weight, both under the RDI (13.48% and 12.40%, respectively) and rainfed (14.21% and 13.11%, respectively) conditions; MC16 also returned the lowest mean berry weight (0.96 g under RDI and 0.78 g under rainfed conditions). All genotypes maintained a mean berry weight under 1.80 g (a quality criterion used in the initial selection process) under both irrigation treatments.

### 2.3. Characterization of the Bunches and Berries

Table 3 shows that the values for most of the variables used in the characterization of the bunches and berries varied significantly (*p* < 0.001) between the genotypes (G), irrigation treatments (T), and years of study (Y), and in terms of the influence of the interaction G × T and G × Y. The interaction of G × T × Y was strong (*p* < 0.001) only for the berry width. MC16, MC19, and MC72 showed the least compact clusters under both irrigation treatments, coinciding with a greater cluster length and shorter berry length and width (Table 3).

The mean length and width of both the bunches and the berries were significantly reduced under rainfed conditions compared to RDI, coinciding with a reduction in the bunch and berry mean weight (Table 2). The mean reduction was 11% for the bunch length, 13% for the bunch width, 9% for the berry length, and 8% for the berry width. Under rainfed conditions, MC19 and MS49 showed a reduction in bunch compactness, coinciding with one of the greatest reductions in bunch length (12% and 18%, respectively) and width (19% and 18%, respectively), and with one of the largest reductions in berry length (10% and 15%, respectively) and width (8% and 15%, respectively).

No variation was seen among the genotypes or irrigation treatments in terms of berry skin and pulp color pulp, or berry flavor. All had a blue-black skin color (OIV 225 rank 9), showed an absence of anthocyanin pigmentation in the pulp (OIV 231 rank 1), and had a flavor catalogued as not moscatel, foxé, or herbaceous (OIV 236 rank 5).

### 2.4. Grape Quality

The mean values for all the variables used to characterize grape quality (Table 4) varied significantly (*p* < 0.001) among the genotypes (G), but only some varied significantly between the irrigation treatments (T) and year of study (Y). Only the interaction G × Y and G × T × Y had any significant influence on all these variables (*p* < 0.001). Both the TPC skin–seed and the anthocyanin contents were significantly higher under the rainfed than the RDI conditions, with a mean increase (period 2018–2021) of 16% and 10%, respectively. Under rainfed conditions, the greatest percentage increase in TPC skin–seed was for MS49 (47%), while MC72 showed the lowest (7%). Except for MC72, which remained in ‘quality group’ 2 (based on mean TPC skin–seed values), a trend was observed for the quality group to improve under these conditions, especially for MS49 (Table 4). The greatest percentage increase in the anthocyanin content was again seen for MS49 (35%), while MC80 had the lowest percentage increase (3%). MC16 and MC80 fell into the best “quality groups” (for both TPC skin–seed and anthocyanin content) under both the RDI and rainfed conditions.

The mean values of parameters such as °Baumé, pH, total acidity and tartaric acid content (period 2018–2021) varied significantly (*p* < 0.001) among the genotypes (G) and year of study (Y), and in terms of the influence of the interaction G × Y and G × T × Y. However, it did not vary significantly with respect to irrigation treatment (Table 4). MS104 reached physiological maturity with the lowest °Baumé value under both the RDI (10.7) and rainfed conditions (10.5) (Table 4) (period 2018–2021). In contrast, MC16 was harvested with the highest °Baumé value (14.2 under both RDI and rainfed conditions.

The mean pH values of MC19, MC80, and MS104 were below the overall mean value under both the RDI and rainfed conditions (Table 4). Moreover, MC80 and MS104 maintained a pH below pH 3.9 in both treatments; this is an initial quality requirement for the pre-selection of genotypes and is of great interest for the production of quality wines in the area).

MC16 had the highest total acidity value (g/L, tartaric acid) under the RDI (4.51) and rainfed (4.90) conditions. In contrast, MC19 had the lowest (3.64 and 3.51, respectively). Although the mean total acidity was slightly higher under the rainfed conditions than under RDI, MC72 and MS104 had significantly lower mean values under the former (3% and 10% lower, respectively). In contrast to the total acidity, the mean tartaric acid content (g/L) tended to be lower under rainfed conditions, except in MC80, which saw an increase of 8% compared to under the RDI condition, although this was not statistically significant (Table 4). MC72 had the highest tartaric acid content (g/L) under the RDI (5.71) and rainfed (5.47) conditions; MC80 had the lowest under RDI (4.51), and MS49 had the lowest under the rainfed conditions (4.72).

The mean malic acid content (period 2018–2021) varied significantly (*p* < 0.001) among the genotypes (G), and in terms of the influence of the interaction G × Y and G × T × Y. It also differed (*p* < 0.01) among irrigation treatments (T) and in terms of the influence of the interaction G × T, but it did not vary significantly between study years (Table 4). MC16 had the highest malic acid content (g/L) under RDI (2.83) and rainfed (2.78) conditions. In contrast, MC80 had the lowest content under RDI (1.51), and MC19 (1.25) under the rainfed conditions. The mean malic acid content was significantly lower under the rainfed than the RDI conditions, particularly for MC19 (22%) and MS104 (27%). The mean tartaric/malic ratio was lowest in MC16—probably explaining it having the highest total acidity value—and was highest in MC19—probably explaining it having the lowest total acidity value.

The maturity index (MI) at the time of harvest, expressed as the relationship °Baumé/total acidity, was estimated for each genotype and irrigation treatment (Table 4). The mean MI (period 2018–2021) varied significantly (*p* < 0.001) among the genotypes (G), and in terms of the influence of the interaction G × Y and G × T × Y. Differences (*p* < 0.01) were also seen with respect to the year of study (Y), but not between the irrigation treatments (Table 4). MS104 had the lowest mean MI under the RDI (2.40) and rainfed conditions (2.62), while MC19 had the highest under RDI (3.77), and MC72 the highest under the rainfed conditions (3.77). The MC19 and MC72 genotypes had values above the overall mean under both treatments, unlike MC16, MS104 and MS49. In general, these findings indicate that higher MI values are more related to a reduction in total acidity than to an increase in the °Baumé value.

### 2.5. Vine Water Status

**Figure 1:**
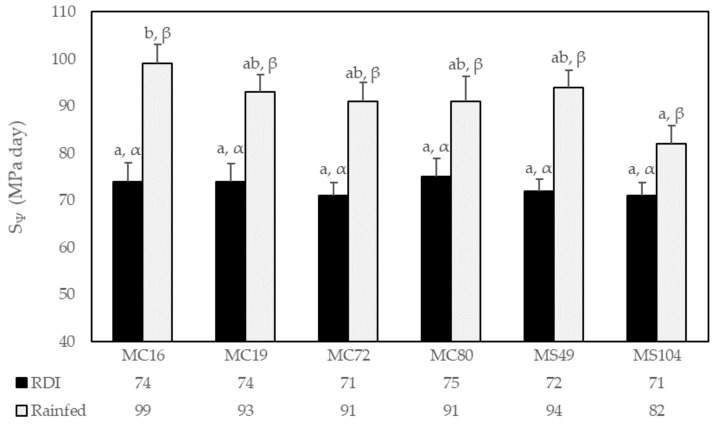
Mean annual water-stress integral (S_Ψ_) values for each genotype and irrigation treatment. Vertical bars represent the standard error. RDI, regulated deficit irrigation. For each irrigation treatment, different letters (a–b) indicate significant differences among genotypes (Duncan’s multiple range test, *p* < 0.05). For each genotype, different letters (α, β) indicate significant differences between irrigation treatments (Duncan’s multiple range test, *p* < 0.05).

The cumulative water stress, calculated as S_Ψ_ (Figure 1), was significantly higher under the rainfed conditions (mean 86 MPa day) than under RDI (mean 69 MPa day), with a mean increase of 26% compared to the latter. MC16 showed the greatest increase (34%) compared to under RDI, and MS104 the lowest (16%). Differences among the genotypes were significant only under the rainfed conditions, ranging from the lowest value of 82 MPa day returned by MS104, to 99 MPa day returned by MC16. A negative correlation was detected between S_Ψ_ and the yield and quality characteristics (*p* < 0.05), both under the RDI and rainfed conditions, i.e., between the S_Ψ_ and the total yield (r = −0.26 and r = −0.37), weight of the bunches (r = −0.18 and r = −0.35), and number of bunches (r = −0.29 and r = −0.25) (probability level *p*< 0.001). In contrast, the S_Ψ_ correlated positively with the anthocyanin content under both the RDI and rainfed conditions (r = 0.22 and r = 0.24), and TPC (r = 0.36 and r = 0.31, for a probability level of *p* < 0.001).

## 3. Discussion

The results presented in this work are of great importance given the risk that the current semi-arid wine-growing areas could undergo in the near future, mainly due to the extreme scarcity of water and high temperatures expected [43], which could cause the exclusion of these areas for wine production [47]. Most of the varieties grown in these areas are adapted to the current conditions of drought and high temperatures, but they may not resist a climate that is drier and warmer as expected. Hence, the importance of obtaining and selecting new plant material that can adapt to these new adverse climatic conditions, maintaining adequate production and good quality in these growing areas. The phenotypic variability found among the six new genotypes studied has allowed us to identify those that could better adapt to the new climate scenario in semi-arid zones.

Some of the phenological and grape quality variables (such as °Baumé, pH and acidity) measured in this work were not significantly affected by the tested irrigation treatments. In contrast, all the productive and morphological variables measured, and those related to phenolic content, were significantly affected (under rainfed conditions the values for productive variables decreased, while those referring to phenolic content increased). Notable variation around the mean change in value was also seen for the different genotypes studied.

In grapevines, the phenology of the plant determines the production window and influences the ability to adapt to climate change [52,53]. One way to adapt vineyards to drought conditions and high temperatures, such as those found in the study area, is the cultivation of late-ripening varieties; this would avoid plants suffering high temperatures during the ripening period. Alternatively, varieties with longer ripening periods or that ripen slowly could be used. Of the six genotypes studied, MC16 and MC80 were the slowest and latest to ripen. Therefore, they could be good candidates for cultivation under hot and dry conditions.

The present results confirm the negative effect of water stress on total yield reported by other authors [26,39,54], and agree with the associated high skin/pulp ratios reported [55,56]. They also confirm that different genotypes show different sensitivities to water scarcity [57,58]. Thus, under rainfed conditions, MS104 was the most productive genotype (34% higher than the least productive) and returned the highest bunch and berry weights. On the other hand, compared to that recorded under RDI conditions, in the present work, MC80 showed the least reduced yield, bunch, and berry weights under rainfed conditions.

There is a relationship between the high phenolic content of the berry and wine quality attributes such as aroma, color, body, etc., particularly in red wines [59,60]. In this regard, under rainfed conditions, MC80 showed the highest TCP skin–seed content and MS49 the highest anthocyanin content, exceeding those of MC72 (which had the lowest contents) by 61% and 79%, respectively. Although phenolic quality is associated with a reduction in production and a smaller berry size [27,61], the present results also show a strong genotypic component since the differences in phenolic variables did not always coincide with differences in the yield variables. For example, the lowest production and the smallest berry size of MC72 and MC19 (Table 2) did not correlate with the highest TCP skin–seed content (Table 4), as might be expected, while the highest production of MS104 did correlate with one of the lowest TCP skin–seed contents. Based on these results, new experiments will be designed to evaluate and confirm the highest quality of wine obtained from the varieties with the highest phenolic content.

In general, when vines have adequate availability of water, their sugar content, must pH, and total acidity will be higher than under conditions of water stress [62]; higher values for these variables were therefore expected under RDI than under the rainfed conditions. However, water availability seemed to have no significant effect on these variables, in agreement with that reported by other authors for total acidity [63], pH [64,65,66], and sugars [66,67,68,69]. Nevertheless, differences were seen at the genotype level with respect to these variables, allowing, for example, for the selection of genotypes such as MS104 with a lower berry sugar content at harvest for use in making wines with a lower alcohol content. This is important given the increase in the accumulation of sugars that normally occurs with rising temperatures, as well as rising consumer demands for low-alcohol wines.

The malic acid content was significantly lower under the rainfed than under the RDI conditions, as reported by other authors [70,71,72,73]. It is known that if water stress intensifies, malic acid is metabolized more [74], while tartaric acid values remain more stable [75]. This might explain why the Tar/Mal ratio was significantly higher under the rainfed conditions, further confirming that the lower acidity recorded in some genotypes is mainly due to their lower malic acid content. Acidity is essential in wine, both from the point of view of its conservation and its organoleptic properties, so a reduction in total acidity, and in particular malic acid, can lead to unbalanced and flat wines [68,76,77]. For this reason, in hot climates, it is necessary to make acidity corrections during fermentation to guarantee the conservation and good evolution of the wine over time. In this regard, under rainfed conditions, MC16 and MC80 showed the highest total acidity, exceeding that of MC19 (which had the lowest total acidity) by 40% and 20%, respectively.

The year of measurement had a significant effect on most of the studied variables, with some exceptions such as TPC skin–seed, malic acid, and the Tar/Mal ratio. The earliest ripening occurred in 2020—the year with the highest maximum temperature during the ripening period (veraison–harvest). This effect was particularly noticeable in the earlier maturing genotypes MC19, MC72, and MS104.

Daytime temperatures > 35 °C during the period from flowering to the beginning of veraison can lead to reduced yields since both flowering and fruit setting are affected [32], and in the present work, such temperatures were recorded for 7 days during this period in 2018, for 10 days in 2019, for 11 days in 2020, and for 9 days in 2021. This may have influenced the reduction in yield and the mean bunch weight recorded in 2019 and 2020.

After the harvest, the plant accumulates reserves for the following year and produces the reproductive meristems responsible for the following year’s production [78]. The present results show that in 2020, during this phase, there was more rainfall than in previous years of study, which might explain the increase in the total yield, bunch weight, and berry weight recorded in 2021.

High temperatures have been correlated with a greater synthesis of anthocyanins and sugars, and with a reduction in acidity [33,34], and the present results show that in 2020—the year with the warmest ripening period—there was an increase in the synthesis of anthocyanins and in the °Baumé value, while the content of tartaric acid was reduced. Nevertheless, despite the effect of the year, the particular behavior of the genotypes was generally maintained over the different years. For example, in most years, MC80 was among the genotypes with the latest harvest dates and one of the genotypes in which the contribution of the skin to the weight of the berry was greatest. It also had among the highest TPC skin–seed and anthocyanin values in most years. MS104 was one of the most productive genotypes, reached physiological maturity with the lowest °Baumé value, and, along with MC80, had one of the lowest must pH values.

## 4. Conclusions

Starting from the premise that genotypes that behave better under rainfed conditions should be those that can best adapt to the effects of climate change in semi-arid areas, and taking into account that temperatures above 35 °C can reduce the yield, total acidity, and phenolic quality and increase the must pH and sugar content (and, therefore, the wine alcohol content), MC80 and MS104 would appear to be candidates for cultivation as climate change takes hold. MC80 suffered below-average water stress, fell into the best “phenolic quality group” for TPC and anthocyanins, and maintained very adequate yield, pH, and total acidity values. MS104 suffered the least water stress and returned the highest yields while maintaining very adequate anthocyanin, pH, and total acidity values. MS104 also had the lowest °Baumé value, rendering it of interest for the production of low-alcohol wines. This genotype might satisfy the requirements of winemakers who seek to produce such wines in hot climates.

The effect of controlled deficit irrigation and drought on other variables, such as leaf area, gas exchange, and wine quality, is now being examined. Having more complete information will aid in our understanding of grapevine responses to drought and high temperatures, and in the selection of the genotypes best adapted to them.

## 5. Materials and Methods

### 5.1. Location and Climate

The plant material used in the present work was cultivated in El Chaparral (Cehegín, Murcia, SE Spain) at the IMIDA’s “Hacienda Nueva” experimental farm (38°06′40.7″ N; 1°40′50.3″ W; altitude 433 m). This site is located in one of the warmest wine-producing areas of the region of Murcia, with hot summers (daily maximum temperatures can exceed 40 °C) and low rainfall (perhaps <350 mm per year). Supplementary Table S1 shows the values for the different meteorological variables—reference evapotranspiration (ETo, mm), precipitation (mm), vapor pressure deficit (VPD, KPa), daily maximum (T_MAX_, °C), average (T_MED_, °C), minimum (T_MIN_, °C) air temperature, cumulative radiation (RAD_CUM_, MJ/m^2^), maximum radiation (RAD_MAX_, W/m^2^), and mean radiation (RAD_MEAN_, W/m^2^)—recorded during the crops’ different phenological periods for the four years of the present study (2018–2021). These variables were monitored daily at a meteorological station (Campbell mod. CR 10 X) belonging to the Murcia Agricultural Information Service (SIAM, http://siam.imida.es/ (accessed on 25 January 2022)), located on the experimental farm. Over 2018–2021 period, a mean annual ETo of 1125 mm was recorded, along with a mean annual rainfall of 384 mm and a mean annual atmospheric VPD of 1.16 KPa.

### 5.2. Plant Material

The plant material used in this study included six new genotypes selected from crosses between “Monastrell” (M) and “Cabernet Sauvignon” (C), and between “Monastrell” (M) and “Syrah” (S): MC16, MC19, MC72, MC80, MS49, and MS104. All genotypes were unequivocally identified (Supplementary Table S2) via PCR and the analysis of eight simple sequence repeat (SSR) markers, as described by Bayo-Canha et al. (2012) [79]. In 2016, scions were grafted onto 110-Ritcher rootstocks planted in 2015; this is the rootstock most commonly used in the area since it shows good adaptation to drought and promotes good grape quality [1,80]. The assessed genotypes were 2 years old at the start of the study, and 5 years old at the end.

The six new genotypes were initially classified into “quality groups”, according to the content of total phenolic compounds in the skins and seeds (TPC skin–seed), and anthocyanins in the skin (Supplementary Table S3; [48,49]). All were selected for their phenolic quality—which was very superior to that of the parentals (Table 5)—based on data obtained over 2012–2017 from the analysis of 20 plants per genotype (on 110-Richter rootstocks), cultivated under sustained deficit irrigation at 40–60% of crop evapotranspiration (ETc) throughout the growing season. The TCP skin–seed and anthocyanin contents were >3100 mg kg^−1^ berry and >2200 mg kg^−1^ berry, respectively, for all six genotypes (Table 5). This exceeds the values for the reference variety of the area ‘Monastrell’ (1528 mg kg^−1^ berry and 939 mg kg^−1^ berry, respectively), as well as for ‘Cabernet Sauvignon’ (2220 mg kg^−1^ berry and 1450 mg kg^−1^ berry, respectively) and ‘Syrah’ (1984 mg kg^−1^ berry and 1583 mg kg^−1^ berry, respectively), all of which are well adapted to the warm climate of the area. MC16, MC80, MS49 and MS104 gave yields similar to or slightly higher than those obtained with ‘Monastrell’ (2.00 kg vine^−1^), while MC19 and MC72 returned the lowest yields (below that of Syrah at 1.59 kg vine^−1^, the least productive parental under rainfed conditions).

### 5.3. Experimental Design and Irrigation Treatments

A randomized block design was followed with two irrigation treatments and three replicates per genotype, irrigation treatment, and parameter evaluated. Each replicate involved six vines per genotype and treatment, of which the outside plants in each row were discounted to avoid potential edge effects. Thus, for each genotype, 24 plants were studied, 12 for each irrigation treatment and parameter evaluated (4 plants per replicate). The training system used was a bilateral cordon trellis with a vertical three-wire system. The rows had a N-NW to S-SE orientation. The distance between rows was 2.5 m, and that between vines was 1 m. The vines were pruned to six two-bud spurs (12 nodes).

The two irrigation treatments were: (1) regulated deficit irrigation (RDI), which contributed 25–30% of the ETc; and (2) rainfed, in which the only water received was from rainfall. This particular RDI treatment was selected since it maintains adequate yields and allows for very good enological quality in the area [39]. Supplementary irrigation (equivalent to the mean historical rainfall of the area for the last 10 years) was allowed for both treatments when necessary to avoid irreversible damage due to very severe water stress (Ψ_S_ < −1.6 MPa): this was needed twice in 12 and 30 August 2018, twice in 6 and 20 August 2019, and once in 22 August 2020; in 2021, it was not required. This covered the entire plot to maintain the total water difference between the treatments. The ETc was calculated as described in Romero et al. (2018) [39].

The irrigation system consisted of two irrigation lines for each row of vines. One of these irrigation lines contained one self-compensating dripper per plant (flow rate of 4 l/h) for applying fertilizer treatments and supplementary irrigation. The other line had 8 l/h self-compensating drippers for use in the RDI treatment, but no drippers for the rainfed rows. In 2015, 2016, and 2017, irrigation (252 mm/plant per year) was applied for the correct establishment of the plot. In 2018, 2019, 2020, and 2021, the experimental irrigation treatments were applied between April and October (sprouting to post-harvest), with an average 143 mm/plant provided per year under the RDI conditions. The cultivation techniques—fertilizer use, phytosanitary treatments, and soil maintenance—were the same throughout the experimental plot. Weed removal was carried out using herbicides in the dripper line and, in the lanes between the rows, using agricultural machinery.

### 5.4. Vines Water Status

The water potential of the stem (Ψ_S_) was determined fortnightly at noon (12:00–13:30 solar hour) from mid-May–June to the end of September–October, using a Model 600 pressure chamber (Soil Moisture Equipment, Santa Barbara, CA, USA). For each genotype and irrigation treatment, four mature, healthy, fully exposed, and expanded leaves located on the main shoots of the upper-middle part of the canopy were selected. These leaves were covered with totally airtight aluminum foil bags for at least 2 h before taking measurements.

The cumulative effect of the water deficit was determined as the water-stress integral (S_Ψ_) calculated, as defined by Myers (1988) [81], as the sum of the mean difference between two consecutive measurements of water potential (${\bar{\Psi }}_{i,i+1}$) and the maximum (least negative) value recorded during the study period$\left(c\right)$, multiplied by the number of days in the interval between one measurement and the next (n) (1).

(1) $$S\Psi =\overset{i=t}{\underset{i\;=0}{\sum }}\left({\bar{\Psi }}_{i,i+1}-c\right)n$$

### 5.5. Phenotypic Evaluation

#### 5.5.1. Phenological Characteristics

During 2018, 2019, 2020 and 2021, the dates for the different phenological stages—budbreak, flowering, veraison, and harvest—for each genotype and irrigation treatment were recorded [82]. In 2019, 2020 and 2021, the dates for the beginning of leaf fall and its completion were also recorded. The date of budbreak was considered as that on which 50% of the buds on a plant were in Baggiolini phenological stage C (green tip); the date of flowering as that on which 50% of the flowers were in phenological stage I (visible stamens); the veraison date as that on which 50% of the berries had started to change color and/or showed a loss of chlorophyll and softening had started (phenological stage M); the date of harvest (phenological stage N) as that on which appropriate physiological maturity had been reached; the date of the start of leaf fall as that on which 5% of the leaves fell (phenological state O1); and the date of total leaf fall as that on which leaf fall was complete (phenological state O2). Physiological maturity was deemed to begin when the grape reached its maximum size and its highest concentration of sugars. At this point, the berry begins to decrease in size due to water loss and some dehydrated berries appear in the cluster, the organoleptic maturity of the skin is good, and the seeds are mature (brown color).

#### 5.5.2. Productive and Morphological Characteristics

For each genotype, irrigation treatment, and replicate (4 plants per replicate), the productiveness and morphological characteristics of representative bunches were assessed at the time of harvest. The yield was recorded as the total number of bunches per plant, total yield (kg/plant), and mean bunch weight (total yield/number of bunches); the mean berry weight was calculated from the weight of 100 randomly selected berries. The morphological characterization of the bunches was performed based on the bunch length (mm), bunch width (mm), and bunch compactness as per OIV 204 descriptors (1, very loose; 3, loose; 5, medium; 7, compact; 9, very compact) [83]. Morphological characterization of the berries was performed using 30 representative berries per replicate and treatment (randomly selected from the different areas of the representative bunches) and based on the berry length (mm) and width (mm) as measured with a Mitutoyo CD-15D digital caliper.

### 5.6. Grape Quality

The grape quality (for each genotype, irrigation treatment, and replicate) was assessed at the IMIDA experimental winery. For each replicate and irrigation treatment, 350 berries were randomly selected from the different areas of the bunches. From this representative sample, 30 berries were taken for the extraction and analysis in triplicate of the TPC skin–seed (mg/kg berry), and of the total anthocyanins (mg/kg berry), as described by Rustioni et al. (2014) [84]. The rest of the berry sample (320 berries) was crushed, without breaking the seed, and centrifuged. The °Baumé value (OIV-MA-AS2-02), total acidity (OIV-MA-AS313-01), must pH (OIV-MA-AS313-15), tartaric acid content (following the modified Rebelein method [85]), and malic acid content (OIV-MA-AS313-11) were analyzed in the must obtained by centrifugation, adhering to the protocols described by Fernández-Fernández et al. (2020) [49]. The grape maturity index (MI) was calculated as the ratio between the °Baumé value and the total acidity. All analyses were performed using randomly selected berries from each replicate per genotype and irrigation treatment.

### 5.7. Statistical Analysis

The collected data were subjected to analysis of variance (three-way ANOVA), using the genotype, irrigation treatment, and year as factors. Means were compared using Duncan’s multiple range test (*p* < 0.05). The correlation between S_Ψ_ and the productiveness and quality traits was calculated using the Spearman test (*p* < 0.05). All analyses were performed using StatGraphics Centurion XVI v.16.1.18 software (StatGraphics Technologies, Inc., The Plains, VA, USA).

## Figures and Tables

**Table 1:** Mean data (2018–2021) for the phenological stage dates of the six new genotypes grown under regulated deficit irrigation (RDI) and rainfed conditions.

	Budbreak		Flowering		Veraison		Harvest		Leaf Fall Start		Total Leaf Fall	
**Genotype**	RDI	Rainfed	RDI	Rainfed	RDI	Rainfed	RDI	Rainfed	RDI	Rainfed	RDI	Rainfed
MC16	Apr 16 ab,α	Apr 16 ab,α	May 28 ab,α	May 29 a,α	Aug 09 c,α	Aug 10 c,α	Sep 14 b,α	Sep 11 c,α	Oct 13 b,α	Oct 13 b,α	Nov 27 c,α	Nov 29 cd,α
MC19	Apr 21 c,α	Apr 24 c,α	Jun 03 c,α	Jun 04 b,α	Aug 04 b,α	Aug 06 b,α	Aug 29 a,α	Sep 05 b,α	Oct 30 d,α	Oct 27 cd,α	Dec 03 d,α	Dec 02 cd,α
MC72	Apr 12 a,α	Apr 12 a,α	May 27 a,α	May 28 a,α	Aug 01 a,α	Aug 02 a,α	Aug 26 a,α	Aug 25 a,α	Nov 08 e,α	Nov 06 e,α	Dec 06 d,α	Dec 05 d,α
MC80	Apr 19 bc,α	Apr 19 bc,α	Jun 01 bc,α	Jun 01 ab,α	Aug 12 d,β	Aug 10 c,α	Sep 13 b,α	Sep 13 c,α	Nov 03 de,α	Nov 02 de,α	Nov 27 c,α	Nov 27 c,α
MS49	Apr 16 b,α	Apr 16 ab,α	May 29 ab,α	May 28 a,α	Jul 31 a,α	Aug 02 a,α	Aug 30 a,α	Aug 29 a,α	Sep 19 a,β	Sep 15 a,α	Nov 09 a,α	Nov 08 a,α
MS104	Apr 16 b,α	Apr 17 ab,α	May 30 abc,α	Jun 01 ab,α	Aug 09 c,α	Aug 11 c,α	Sep 09 b,α	Sep 06 b,α	Oct 21 c,α	Oct 20 bc,α	Nov 22 b,α	Nov 20 b,α
**Irrigation**	Apr 17 α	Apr 18 α	May 30 α	May 31 α	Aug 06 α	Aug 07 α	Sep 04 α	Sep 04 α	Oct 21 α	Oct 20 α	Nov 26 α	Nov 26 α
**Year**												
2018	Apr 11 a,α	Apr 11 a,α	May 27 a,α	May 26 b,α	Aug 04 a,α	Aug 06 ab,α	Sep 10 b,α	Sep 14 c,α	-	-	-	-
2019	Apr 25 c,α	Apr 27 c,α	Jun 08 b,α	Jun 10 c,β	Aug 07 a,α	Aug 09 b,β	Sep 04 ab,α	Sep 04 b,α	Oct 31 b,α	Oct 31 b,α	Nov 30 b,α	Nov 29 b,α
2020	Apr 13 a,α	Apr 13 a,α	May 26 a,α	May 25 a,α	Aug 06 a,α	Aug 05 a,α	Aug 30 a,α	Aug 30 a,α	Oct 14 a,α	Oct 12 a,α	Nov 26 b,α	Nov 27 b,α
2021	Apr 16 b,α	Apr 16 b,α	May 27 a,α	May 28 b,α	Aug 06 a,α	Aug 05 a,α	Sep 06 b,α	Sep 03 b,α	Oct 18 a,α	Oct 17 a,α	Nov 21 aα	Nov 19 a,α

For each genotype and year, different letters in the same column (a–e) indicate significant differences among genotypes and years, respectively, at the 5% level, according to Duncan’s multiple range test. For each phenological stage date, different letters in the same row (α, β) indicate significant differences between the irrigation treatments (Duncan test, *p* < 0.05).

**Table 2:** Yield components of the six genotypes grown under regulated deficit irrigation (RDI) and rainfed conditions over the four-year study period (2018–2021), and the mean values for that period.

		2018		2019		2020		2021		2018–2021		ANOVA						
	**Genotype**	RDI	Rainfed	RDI	Rainfed	RDI	Rainfed	RDI	Rainfed	RDI	Rainfed	G	T	Y	G × T	G × Y	T × Y	G × T × Y
Yield (kg vine-1)	MC16	2.55 b,α	2.10 b,α	1.97 bc,β	0.86 ab,α	1.12 a,α	0.68 ab,α	2.29 ab,α	1.53 ab,α	1.94 a,β	1.38 ab,α	***	***	***	*	**	ns	ns
	MC19	1.83 a,β	1.56 a,α	1.10 a,α	0.50 a,α	1.29 a,β	0.63 ab,α	2.43 abc,β	1.32 ab,α	1.76 a,β	1.03 a,α							
	MC72	1.75 a,β	1.25 a,α	1.50 abc,β	0.54 a,α	1.82 b,β	0.83 ab,α	1.84 ab,β	1.24 ab,α	1.76 a,β	1.03 a,α							
	MC80	2.89 bc,β	2.07 b,α	1.30 ab,α	0.77 ab,α	1.04 a,α	0.79 ab,α	1.78 a,α	1.51 ab,α	1.77 a,β	1.39 ab,α							
	MS104	2.90 bc,β	2.00 b,α	2.18 c,α	1.33 b,α	1.39 ab,α	0.92 b,α	3.47 c,β	1.70 b,α	2.52 b,β	1.51 b,α							
	MS49	3.17 c,β	2.27 b,α	2.03 bc,β	0.62 ab,α	1.34 ab,β	0.42 a,α	2.95 bc,β	0.90 a,α	2.47 b,β	1.14 ab,α							
	**Average**	**2.54 β**	**1.88 α**	**1.67 β**	**0.74 α**	**1.33 β**	**0.71 α**	**2.40 β**	**1.39 α**	**2.03 β**	**1.23 α**							
Nº bunches vine-1	MC16	20 a,α	20 a,α	22 bc,α	23 c,α	19 a,α	21 c,α	19 a,α	19 b,α	20 a,α	20 bc,α	***	***	***	ns	***	**	*
	MC19	22 a,α	27 b,α	13 a,α	10 a,α	19 a,β	12 a,α	18 a,α	13 a,β	19 a,β	15 a,α							
	MC72	25 ab,α	22 ab,α	21 bc,α	15 b,α	23 b,β	18 bc,α	21 a,β	18 b,α	23 b,β	18 abc,α							
	MC80	22 a,α	21 a,α	21 bc,α	17 bc,α	17 a,α	15 ab,α	19 a,α	17 ab,α	19 a,α	17 ab,α							
	MS104	22 a,α	20 a,α	17 ab,α	19 bc,α	18 a,α	14 ab,α	18 a,α	17 b,α	19 a,α	17 ab,α							
	MS49	28 b,α	33 c,β	23 c,α	17 b,α	20 ab,β	16 abc,α	22 a,α	16 ab,β	23 b,α	22 c,α							
	**Average**	**23 α**	**24 α**	**20 β**	**16 α**	**19 β**	**16 α**	**20 β**	**16 α**	**21 β**	**18 α**							
Bunch weight (g)	MC16	130.90 c,α	108.97 b,α	88.59 a,β	37.15 a,α	58.03 a,β	30.10 ab,α	115.20 ab,α	79.60 abc,α	97.60 ab,β	69.21 bc,α	***	***	***	*	***	ns	ns
	MC19	89.05 ab,β	62.70 a,α	80.21 a,β	43.56 a,α	67.26 ab,β	45.66 bc,α	130.05 b,β	88.12 bc,α	93.53 ab,β	61.79 abc,α							
	MC72	70.35 a,α	61.84 a,α	69.46 a,β	34.78 a,α	78.40 b,β	46.66 bc,α	86.38 a,β	67.83 ab,α	77.52 a,β	55.54 ab,α							
	MC80	139.49 c,β	99.12 b,α	60.92 a,α	38.41 a,α	62.40 ab,α	44.88 bc,α	92.83 a,α	89.26 bc,α	90.99 ab,α	74.25 cd,α							
	MS104	148.07 c,α	112.31 b,α	124.78 b,β	68.28 a,α	73.39 ab,α	60.49 c,α	176.14 c,β	98.11 c,α	131.04 c,β	87.46 d,α							
	MS49	118.03 bc,β	70.57 a,α	87.14 a,β	36.37 a,α	64.52 ab,β	24.91 a,α	133.74 b,β	56.57 a,α	104.10 b,β	48.93 a,α							
	**Average**	**116.10 β**	**85.36 α**	**84.56 β**	**42.82 α**	**66.82 β**	**42.17 α**	**119.41 β**	**79.38 α**	**98.53 β**	**65.51 α**							
Berry weight (g)	MC16	0.97 b,β	0.75 a,α	0.71 a,β	0.52 a,α	0.85 a,β	0.64 a,α	1.39 b,β	0.93 a,α	0.96 a,β	0.78 a,α	***	***	***	***	***	**	***
	MC19	1.17 c,β	0.79 a,α	0.98 b,β	0.64 ab,α	1.00 ab,α	0.89 bc,α	1.42 b,β	1.03 ab,α	0.99 ab,β	0.86 b,α							
	MC72	0.94 b,β	0.78 a,α	0.97 b,β	0.77 b,α	1.13 b,β	0.83 abc,α	1.17 a,α	1.13 b,α	1.06 ab,β	0.86 b,α							
	MC80	1.17 c,α	0.88 a,α	1.08 b,β	0.66 ab,α	1.04 ab,α	0.96 c,α	1.34 b,α	1.27 c,α	1.09 b,α	1.03 c,α							
	MS104	0.72 a,α	0.70 a,α	1.03 b,α	0.78 b,α	1.19 b,α	1.02 c,α	1.61 c,β	1.34 c,α	1.22 c,β	1.03 c,α							
	MS49	1.20 c,β	0.82 a,α	1.19 b,β	0.59 ab,α	1.19 b,β	0.66 ab,α	1.75 d,β	1.01 a,α	1.39 d,β	0.81 ab,α							
	**Average**	**1.05 β**	**0.88 α**	**1.00 β**	**0.77 α**	**1.00 β**	**0.82 α**	**1.44 β**	**1.12 α**	**1.12 β**	**0.89 α**							
% skin	MC16	18.63 d,β	15.11 c,α	9.98 c,α	11.71 b,α	12.27 b,α	13.49 c,α	11.04 d,α	12.57 c,β	13.48 e,α	14.21 d,α	***	*	***	***	***	ns	***
	MC19	8.66 a,α	12.45 b,β	9.39 c,α	8.77 a,α	08.37 a,α	7.98 a,α	7.72 a,α	8.66 a,β	9.99 b,β	9.03 a,α							
	MC72	13.41 c,β	11.00 b,α	9.60 c,α	9.17 a,α	11.67 b,α	10.13 b,α	10.33 c,α	10.22 b,α	10.72 c,α	11.23 b,α							
	MC80	13.46 c,α	15.46 c,α	12.31 d,α	12.19 b,α	11.96 b,α	12.04 bc,α	12.67 e,α	12.41 c,α	12.40 d,α	13.11 c,β							
	MS104	11.20 b,α	10.99 b,α	7.87 b,α	7.51 a,α	7.10 a,α	6.85 a,α	8.77 b,α	8.70 a,α	9.65 b,α	9.10 a,α							
	MS49	7.31 a,α	8.17 a,α	5.83 a,α	8.26 a,β	7.50 a,α	7.93 a,α	7.89 a,α	9.80 b,β	8.29 a,α	10.87 b,β							
	**Average**	**11.69 α**	**11.27 α**	**9.78 α**	**11.25 β**	**11.25 α**	**11.68 α**	**9.74 α**	**10.39 β**	**10.75 α**	**11.26 β**							
% seeds	MC16	7.51 bcd,α	8.13 ab,α	8.86 b,α	13.87 c,β	8.97 b,α	10.23 c,α	6.89 d,α	9.55 e,β	8.68 d,α	10.88 e,β	***	***	***	***	***	ns	***
	MC19	4.47 a,α	8.38 abc,β	5.73 a,α	7.40 a,α	6.41 a,α	5.61 a,α	3.90 a,α	5.18 a,β	5.27 a,α	6.20 a,β							
	MC72	7.92 cd,α	10.10 c,α	6.66 a,α	8.43 ab,α	8.95 b,α	10.67 c,α	7.09 d,α	7.27 c,α	7.76 c,α	10.30 de,β							
	MC80	5.70 ab,α	6.59 a,α	8.54 b,α	8.96 ab,α	8.43 b,α	10.29 c,α	7.94 e,α	8.52 d,β	9.32 e,α	9.36 c,α							
	MS104	8.39 d,α	9.51 bc,α	7.11 a,α	9.90 ab,β	6.29 a,α	7.84 b,β	5.01 b,α	6.40 b,β	6.57 b,α	7.92 b,β							
	MS49	6.47 bc,α	9.36 bc,β	6.65 a,α	10.08 b,β	7.49 ab,α	9.82 bc,β	5.53 c,α	8.06 d,β	6.63 b,α	9.87 cd,β							
	**Average**	**6.74 α**	**8.68 β**	**7.26 α**	**9.77 β**	**7.76 α**	**9.08 β**	**6.06 α**	**7.49 β**	**7.38 α**	**9.11 β**							

For each productive variable, year and irrigation treatment, different letters in the same column (a–e) indicate significant differences among genotypes (Duncan’s multiple range test, *p* < 0.05). For each productive variable, genotype and year, different letters in the same row (α, β) indicate significant differences between the irrigation treatments (Duncan’s multiple range test, *p* < 0.05). %skin, percentage contribution of the skin to berry weight; %seeds, percentage contribution of the seeds to berry weight. Analysis of variance (three-way ANOVA) by genotype (G), irrigation treatment (T), year (Y) and their interactions: ns: non-significant; * *p* < 0.05; ** *p* < 0.01; *** *p* < 0.001.

**Table 3:** Morphological characterization of the bunches and berries of the new genotypes grown under regulated deficit irrigation (RDI) and rainfed conditions over the four-year study period (2018–2021), and the mean values for that period.

		2018		2019		2020		2021		2018–2021		ANOVA						
	**Genotype**	RDI	Rainfed	RDI	Rainfed	RDI	Rainfed	RDI	Rainfed	RDI	Rainfed	G	T	Y	G × T	G × Y	T × Y	G × T × Y
Bunch lenght (mm)	MC16	173 bc,α	176 c,α	136 ab,α	127 a,α	147 ab,α	156 b,α	162 ab,α	161 c,α	155 bc,α	155 d,α	***	***	***	**	***	ns	*
	MC19	191 c,β	164 bc,α	129 ab,α	125 a,α	167 bc,α	152 b,α	163 ab,β	128 a,α	162 cd,β	142 c,α							
	MC72	174 bc,α	156 abc,α	154 b,β	121 a,α	174 c,α	148 b,α	170 b,β	145 abc,α	168 d,β	143 c,α							
	MC80	156 ab,α	137 a,α	121 a,α	117 a,α	152 abc,β	127 ab,α	150 a,α	153 bc,α	145 ab,β	134 bc,α							
	MS104	132 a,α	130 a,α	128 a,α	111 a,α	144 ab,β	113 a,α	157 ab,α	143 ab,α	140 a,β	124 ab,α							
	MS49	151 ab,α	141 ab,α	139 ab,β	99 a,α	136 a,β	113 a,α	164 ab,β	131 a,α	148 ab,β	121 a,α							
	**Average**	**163 α**	**151 α**	**135 β**	**117 α**	**153 β**	**135 α**	**161 β**	**143 α**	**153 β**	**136 α**							
Bunch width (mm)	MC16	94 abc,α	112 d,β	95 ab,α	79 ab,α	88 ab,α	81 bc,α	87 a,α	86 b,α	91 b,α	90 c,α	***	***	***	***	***	ns	*
	MC19	115 cd,α	97 bcd,α	92 ab,α	78 ab,α	87 ab,α	71 b,α	97 ab,β	70 a,α	98 bc,β	79 b,α							
	MC72	129 d,β	106 cd,α	114 b,β	81 ab,α	119 c,α	103 c,α	128 c,β	102 c,α	123 d,β	98 d,α							
	MC80	100 bc,α	87 ab,α	80 a,α	79 ab,α	89 ab,α	77 b,α	108 b,α	99 c,α	95 bc,β	85 bc,α							
	MS104	85 ab,α	89 abc,α	116 b,α	93 b,α	95 b,α	93 bc,α	109 b,β	95 bc,α	101 c,β	93 cd,α							
	MS49	75 a,α	71 a,α	71 a,α	59 a,α	70 a,β	47 a,α	87 a,β	70 a,α	76 a,β	62 a,α							
	**Average**	**100 α**	**94 α**	**95 β**	**78 α**	**91 β**	**79 α**	**103 β**	**87 α**	**97 β**	**84 α**							
Bunch compactness (OIV)	MC16	5	5	3	3	3	3	5	5	3–5	3–5							
	MC19	5	3	5	3	5	3	7	5	5	3							
	MC72	3	3	1	1	1	1	1	1	1	1							
	MC80	7	7	5	5	7	7	7	7	7	7							
	MS104	7	7	7	7	7	7	7	7	7	7							
	MS49	7	7	7	7	7	3	7	3	7	5							
	**Average**																	
Berry length (mm)	MC16	11.50 bc,α	11.29 c,α	10.49 a,β	9.08 a,α	10.45 a,α	10.44 a,α	11.56 a,β	10.86 a,α	11.00 a,β	10.42 a,α	***	***	***	**	***	ns	**
	MC19	12.16 cd,β	10.59 ab,α	10.83 ab,α	9.77 ab,α	11.05 a,α	10.74 a,α	12.85 b,β	11.17 a,α	11.72 bc,β	10.57 ab,α							
	MC72	10.91 ab,α	10.67 abc,α	11.15 abc,β	10.07 ab,α	12.23 bc,β	10.97 a,α	12.24 ab,α	12.04 b,α	11.63 b,β	10.94 abc,α							
	MC80	12.44 d,β	11.07 bc,α	11.95 c,β	9.74 ab,α	11.86 b,α	11.29 a,α	12.26 ab,α	12.07 b,α	12.13 bc,β	11.04 bc,α							
	MS104	10.59 a,α	10.28 a,α	11.48 bc,α	10.69 b,α	11.95 b,β	11.00 a,α	14.84 c,β	13.13 c,α	12.21 c,β	11.27 c,α							
	MS49	13.95 e,β	12.10 d,α	12.96 d,β	10.35 b,α	12.96 c,β	10.73 a,α	14.20 c,β	12.77 c,α	13.52 d,β	11.49 c,α							
	**Average**	**11.93 β**	**11.00 α**	**11.48 β**	**9.95 α**	**11.75 β**	**10.86 α**	**12.99 β**	**12.01 α**	**12.04 β**	**10.95 α**							
Berry width (mm)	MC16	11.44 b,α	10.89 b,α	10.296 a,β	9.33 a,α	10.70 a,β	9.11 a,α	11.51 a,β	10.62 a,α	10.98 a,β	9.99 a,α	***	***	***	**	***	ns	***
	MC19	12.08 bc,β	10.55 ab,α	10.81 a,α	10.05 ab,α	11.09 ab,α	10.85 b,α	12.55 ab,β	11.38 b,α	11.63 bc,β	10.71 bc,α							
	MC72	10.50 a,α	10.45 ab,α	10.87 ab,β	9.99 ab,α	12.05 c,β	10.61 b,α	11.69 bc,α	11.74 bc,α	11.28 ab,β	10.70 bc,α							
	MC80	12.20 c,β	10.74 b,α	11.64 bc,α	10.11 ab,α	11.61 bc,α	11.49 b,α	12.33 c,α	12.10 cd,α	11.94 cd,β	11.11 c,α							
	MS104	9.93 a,α	10.06 a,α	10.93 ab,α	10.56 b,α	12.06 c,β	11.19 b,α	13.63 c,α	12.60 d,α	11.64 bc,α	11.10 c,α							
	MS49	12.02 bc,β	10.84 b,α	11.89 c,β	9.73 ab,α	12.44 c,β	9.58 a,α	12.48 d,β	11.46 bc,α	12.21 d,β	10.40 ab,α							
	**Average**	**11.36 β**	**10.59 α**	**11.07 β**	**9.96 α**	**11.66 β**	**10.47 α**	**12.37 β**	**11.65 α**	**11.61 β**	**10.67 α**							

For each morphological variable, year and irrigation treatment, different letters in the same column (a–e) indicate significant differences among genotypes (Duncan’s multiple range test, *p* < 0.05). For each morphological variable, genotype and year, different letters in the same row (α, β) indicate significant differences between the irrigation treatments (Duncan’s multiple range test, *p* < 0.05). OIV 204 descriptor (bunch compactness): 1, very loose; 3, loose; 5, medium; 7, compact; 9, very compact. Analysis of variance (three-way ANOVA) by genotype (G), irrigation treatment (T), year (Y) and their interactions: ns: non-significant; * *p* < 0.05; ** *p* < 0.01; *** *p* < 0.001.

**Table 4:** Values of different quality variables for the grapes of the new genotypes grown under regulated deficit irrigation (RDI) and rainfed conditions over the four-year study period (2018–2021), and the mean values for that period.

		2018		2019		2020		2021		2018–2021		ANOVA						
	**Genotype**	RDI	Rainfed	RDI	Rainfed	RDI	Rainfed	RDI	Rainfed	RDI	Rainfed	G	T	Y	G × T	G × Y	T × Y	G × T × Y
TPC skin-seed (mg kg^−1^ berry)	MC16	2849 c,α	3217 b,β	3289 b,α	3764 de,β	3560 d,α	3352 c,β	2915 d,α	3627 c,β	3222 d,α	3504 c,β	***	***	ns	***	***	ns	***
	MC19	2038 a,α	3093 b,β	2432 a,α	3204 c,β	2479 b,α	2485 a,α	2053 a,α	2429 a,β	2271 ab,α	2603 b,β							
	MC72	2098 a,α	3316 b,β	2598 a,β	2101 a,α	2148 a,α	2443 a,β	2340 b,α	2346 a,α	2262 a,α	2415 a,β							
	MC80	3239 d,α	3844 c,β	3214 b,α	3555 d,β	3217 c,α	3999 d,β	3755 e,α	3831 d,α	3444 e,α	3884 d,β							
	MS104	3483 d,β	2462 a,α	2445 a,α	2609 b,α	2356 b,α	2749 b,β	2276 b,α	2674 b,β	2391 bc,α	2688 b,β							
	MS49	2408 b,α	3138 b,β	2371 a,α	4010 e,β	2203 a,α	3222 c,β	2654 c,α	3846 d,β	2421 c,α	3563 c,β							
	**Average**	**2686 α**	**3178 β**	**2725 α**	**3207 β**	**2661 α**	**3042 β**	**2665 α**	**3125 β**	**2662 α**	**3090 β**							
TPC Quality group^¥^	MC16	2–3	3–4	4	4	4	4	3	4	3–4	4							
	MC19	1–2	3	2	3–4	2	2	1–2	2	2	2–3							
	MC72	1–2	4	2–3	2	2	2	2	2	2	2							
	MC80	3–4	4	3–4	4	3–4	4	4	4	4	4							
	MS104	4	2	2	2–3	2	2–3	2	2–3	2	2–3							
	MS49	2	3	2	4	2	3–4	2–3	4	2	4							
	**Average**	**2–3**	**3**	**2–3**	**3–4**	**2–3**	**3**	**2–3**	**3**	**2–3**	**3**							
Anthocyanins (mg kg^−1^ berry)	MC16	2844 d,α	3322 c,β	2637 c,α	3393 d,β	3525 e,α	3473 d,α	2725 c,α	3217 d,β	3059 f,α	3349 e,β	***	***	***	***	***	ns	***
	MC19	2103 b,α	3344 c,β	2724 c,α	2913 c,α	2806 d,α	2815 b,α	2146 b,α	2323 b,β	2483 c,α	2645 c,β							
	MC72	1213 a,α	2254 b,β	1437 a,α	1470 a,α	2108 a,β	2000 a,α	2143 b,α	2056 a,α	2015 a,α	1983 a,α							
	MC80	2807 d,α	3224 c,β	3144 d,α	3268 d,β	2841 d,α	3081 c,β	2947 d,β	2792 c,α	2916 e,α	2999 d,α							
	MS104	2362 bc,β	1935 a,α	2190 b,α	2220 b,α	2463 b,α	2669 b,β	1963 a,α	2127 a,β	2218 b,α	2355 b,β							
	MS49	2385 c,α	3173 c,β	2734 c,α	4115 e,β	2636 c,α	3577 d,β	2618 c,α	3415 e,β	2625 d,α	3544 f,β							
	**Average**	**2286 α**	**2875 β**	**2478 α**	**2897 β**	**2730 α**	**2936 β**	**2424 α**	**2655 β**	**2543 α**	**2796 β**							
Anthoc Quality group^¥^	MC16	4	4	4	4	4	4	4	4	4	4							
	MC19	2–3	4	4	4	4	4	2–3	3	3	4							
	MC72	2	3	2	2	2–3	2–3	2–3	2–3	2–3	2–3							
	MC80	4	4	4	4	4	4	4	4	4	4							
	MS104	3	2–3	2–3	2–3	3	4	2–3	2–3	2–3	3							
	MS49	3	4	4	4	4	4	4	4	4	4							
	**Average**	**3**	**4**	**3**	**4**	**4**	**4**	**3**	**4**	**3**	**4**							
^o^Baumé	MC16	13.8 d,α	13.8 f,α	12.8 b,α	14.2 e,β	14.8 e,α	14.6 d,α	14.2 d,α	13.8 cd,α	14.2 d,α	14.2 e,α	***	ns	**	**	***	ns	***
	MC19	11.6 b,β	11.2 b,α	13.8 e,β	13.6 d,α	14.2 de,α	13.9 c,α	13.2 bc,α	12.4 b,β	13.5 c,α	13.0 bc,α							
	MC72	14.1 e,β	13.3 e,α	14.2 f,β	13.3 b,α	13.6 cd,β	13.1 b,α	14.1 d,α	14.2 d,α	13.9 cd,α	13.5 cd,α							
	MC80	11.5 b,α	12.7 d,β	13.2 d,α	13.4 c,β	12.4 b,α	12.9 b,β	13.5 cd,α	12.0 b,β	12.8 b,α	12.6 b,α							
	MS104	9.4 a,α	10.5 a,β	10.8 a,β	9.0 a,α	11.3 a,α	11.1 a,α	10.3 a,α	10.2 a,α	10.7 a,α	10.5 a,α							
	MS49	12.6 c,β	11.9 c,α	13.1 c,α	15.2 f,β	13.2 c,α	14.4 cd,α	12.5 b,α	13.4 c,β	12.9 b,α	13.8 de,β							
	**Average**	**12.2 α**	**12.2 α**	**13.0 α**	**13.13 α**	**13.2 α**	**13.3 α**	**13.0 α**	**12.7 α**	**13.0 α**	**12.9 α**							
pH	MC16	4.17 d,β	4.08 c,α	3.84 b,α	3.86 c,α	3.97 c,α	4.02 cd,α	4.14 bc,α	4.27 c,β	4.04 d,α	4.11 c,α	***	ns	***	ns	***	ns	***
	MC19	3.95 b,α	3.99 b,β	3.93 c,α	4.02 d,β	3.96 c,α	3.90 b,α	3.94 a,α	3.91 ab,α	3.95 bc,α	3.93 b,α							
	MC72	4.18 d,α	4.14 d,α	4.02 d,α	4.01 d,α	3.85 b,α	4.03 d,β	4.20 c,α	4.23 c,α	4.02 cd,α	4.11 c,α							
	MC80	3.98 c,α	4.10 c,β	3.69 a,β	3.58 a,α	3.64 a,α	3.70 a,α	4.06 b,α	3.96 b,α	3.85 a,α	3.83 a,α							
	MS104	3.82 a,α	3.91 a,β	3.93 c,β	3.69 b,α	3.88 b,α	3.91 bc,α	3.90 a,α	3.85 a,α	3.88 ab,α	3.86 ab,α							
	MS49	4.00 c,α	4.01 b,α	4.03 d,α	4.11 e,β	4.05 d,α	4.08 d,α	3.97 a,α	3.96 b,α	4.01 cd,α	4.03 c,α							
	**Average**	**4.01 α**	**4.04 α**	**3.91 α**	**3.88 α**	**3.89 α**	**3.94 α**	**4.04 α**	**4.03 α**	**3.96 α**	**3.98 α**							
TA (g L^−1^ tartaric)	MC16	4.10 d,α	4.89 c,β	6.11 d,α	6.93 e,β	4.86 e,α	4.93 d,α	3.86 bc,α	4.37 c,β	4.51 c,α	4.90 c,α	***	ns	***	**	***	ns	***
	MC19	3.50 b,β	3.02 a,α	3.92 a,α	4.21 b,β	3.23 a,α	3.46 a,β	4.01 c,β	3.51 a,α	3.64 a,α	3.51 a,α							
	MC72	3.92 c,β	3.56 ab,α	3.91 a,α	3.57 a,α	3.76 b,α	3.68 ab,α	3.59 b,α	3.51 a,α	3.72 a,β	3.59 a,α							
	MC80	3.36 a,α	3.11 a,α	4.98 c,α	5.41 d,β	4.57 d,α	4.65 c,α	3.10 a,α	3.75 a,β	3.90 ab,α	4.21 b,α							
	MS104	4.26 e,α	4.22 bc,α	4.35 b,α	4.90 c,β	4.26 c,β	3.77 b,α	4.84 e,β	4.08 b,α	4.50 c,β	4.05 b,α							
	MS49	3.45 ab,α	4.98 c,β	4.48 b,α	5.26 d,β	3.84 b,α	3.84 b,α	4.49 d,α	4.57 c,α	4.13 b,α	4.39 b,α							
	**Average**	**3.76 α**	**3.96 α**	**4.62 α**	**5.05 α**	**4.09 α**	**4.05 α**	**3.98 α**	**3.96 α**	**4.06 α**	**4.10 α**							
Tar (g L^−1^)	MC16	5.99 d,α	6.85 e,β	4.61 a,α	3.70 a,α	4.20 a,α	4.01 a,α	5.68 c,α	5.48 ab,α	5.01 b,α	4.85 a,α	***	ns	***	ns	***	ns	***
	MC19	5.58 c,β	4.98 bc,α	5.50 c,α	5.61 c,α	5.23 c,α	5.34 d,α	5.92 cd,β	5.66 b,α	5.57 c,α	5.46 b,α							
	MC72	5.62 c,α	5.96 d,α	6.49 d,β	5.44 c,α	5.26 c,β	4.55 bc,α	6.12 d,α	6.51 c,α	5.71 c,α	5.47 b,α							
	MC80	3.98 a,β	3.30 a,α	4.58 a,α	4.59 b,α	4.33 a,α	5.00 d,β	4.81 a,α	5.16 a,α	4.51 a,α	4.85 a,α							
	MS104	5.24 b,α	5.11 c,α	4.93 b,β	4.18 b,α	5.08 bc,α	4.61 c,α	5.80 cd,α	5.78 b,α	5.37 bc,α	5.08 ab,α							
	MS49	5.77 cd,β	4.91 b,α	4.72 a,α	4.61 b,α	4.58 ab,β	4.24 ab,α	5.28 b,α	5.17 a,α	4.99 b,α	4.72 a,α							
	**Average**	**5.36 α**	**5.18 α**	**5.14 α**	**4.69 α**	**4.78 α**	**4.62 α**	**5.60 α**	**5.62 α**	**5.20 α**	**5.08 α**							
Mal (g L^−1^)	MC16	2.83 d,β	2.57 d,α	3.68 f,α	3.85 f,α	2.67 d,α	2.84 c,α	2.77 c,β	2.50 e,α	2.83 c,α	2.78 d,α	***	**	ns	**	***	*	***
	MC19	1.41 a,β	1.07 a,α	1.33 a,α	1.52 b,β	1.28 a,α	1.24 a,α	2.05 b,β	1.23 a,α	1.60 a,β	1.25 a,α							
	MC72	2.61 c,β	1.88 c,α	1.93 c,β	1.65 c,α	1.99 b,α	2.26 b,β	2.21 b,β	1.90 c,α	2.12 b,α	2.04 b,α							
	MC80	1.78 b,α	1.65 b,α	1.70 b,β	1.34 a,α	1.51 a,α	1.40 a,α	1.40 a,α	1.44 b,α	1.51 a,α	1.43 a,α							
	MS104	1.85 b,α	2.04 c,α	2.67 d,β	2.36 d,α	2.30 c,α	2.00 b,α	3.24 c,β	1.74 c,α	2.67 c,β	1.94 b,α							
	MS49	1.76 b,α	2.48 d,β	3.10 e,β	3.06 e,α	2.37 cd,α	2.15 b,α	2.76 c,β	2.26 d,α	2.53 c,α	2.32 c,α							
	**Average**	**2.04 α**	**1.95 α**	**2.40 α**	**2.29 α**	**2.02 α**	**1.98 α**	**2.41 β**	**1.84 α**	**2.21 β**	**1.96 α**							
Tar/Mal	MC16	2.12 a,α	2.66 b,β	1.25 a,β	0.96 a,α	1.59 a,β	1.42 a,α	2.06 a,α	2.22 a,α	1.80 a,α	1.82 a,α	***	*	ns	*	***	*	***
	MC19	3.97 d,α	4.68 d,β	4.14 f,β	3.70 e,α	4.12 e,α	4.34 e,α	2.92 b,α	4.65 c,β	3.63 d,α	4.43 d,β							
	MC72	2.15 a,α	3.17 c,β	3.38 e,α	3.30 d,α	2.67 cd,β	2.06 b,α	2.79 b,α	3.45 b,β	2.73 b,α	2.78 b,α							
	MC80	2.24 a,α	2.01 a,α	2.71 d,α	3.44 d,β	2.94 d,α	3.56 d,β	3.66 c,α	3.74 b,α	3.13 c,α	3.47 c,α							
	MS104	2.84 b,α	2.51 b,α	1.85 c,α	1.78 c,α	2.33 bc,α	2.43 c,α	1.96 a,α	3.35 b,β	2.19 a,α	2.74 b,β							
	MS49	3.29 c,β	1.98 a,α	1.52 b,α	1.51 b,α	1.97 ab,α	1.99 b,α	1.92 a,α	2.30 a,β	2.03 a,α	2.06 a,α							
	**Average**	**2.77 α**	**2.84 α**	**2.47 α**	**2.44 α**	**2.60 α**	**2.63 α**	**2.55 α**	**3.28 β**	**2.59 α**	**2.88 β**							
MI	MC16	3.36 bc,β	2.82 a,α	2.09 a,α	2.05 b,α	3.04 b,α	2.98 a,α	3.69 cd,β	3.17 b,α	3.24 b,β	2.95 b,α	***	ns	**	ns	***	ns	***
	MC19	3.31 b,α	3.72 b,β	3.52 e,β	3.23 e,α	4.40 d,β	4.02 c,α	3.31 c,α	3.57 c,α	3.77 c,α	3.73 c,α							
	MC72	3.59 d,α	3.75 b,α	3.65 f,α	3.73 f,α	3.62 c,α	3.56 b,α	3.94 d,α	4.05 d,α	3.74 c,α	3.77 c,α							
	MC80	3.43 c,α	4.10 b,β	2.65 c,β	2.47 c,α	2.72 a,α	2.78 a,α	4.40 e,β	3.25 bc,α	3.46 bc,α	3.07 b,α							
	MS104	2.22 a,α	2.49 a,β	2.50 b,β	1.84 a,α	2.65 a,α	2.95 a,β	2.17 a,α	2.52 a,β	2.40 a,α	2.62 a,β							
	MS49	3.66 d,α	2.43 a,α	2.92 d,α	2.89 d,α	3.45 c,α	3.77 b,α	2.81 b,α	2.95 b,α	3.16 b,α	3.22 b,α							
	**Average**	**3.26 α**	**3.22 α**	**2.89 α**	**2.70 α**	**3.31 α**	**3.34 α**	**3.38 α**	**3.25 α**	**3.30 α**	**3.24 α**							

TPC, total phenolic content in skin and seed; TA, total acidity; Tar, tartaric acid; Mal, malic acid; Tar/Mal, ratio of tartaric acid to malic acid; MI, maturity index expressed as the ratio of the °Baumé value to total acidity. ¥: classification according to the values shown in Supplementary Table S3. For each quality variable, year and irrigation treatment, different letters in the same column (a–f) indicate significant differences among genotypes (Duncan’s multiple range test, *p* < 0.05). For each quality variable, genotype and year, different letters in the same row (α, β) indicate significant differences between the irrigation treatments (Duncan’s multiple range test, *p* < 0.05). Analysis of variance (three-way ANOVA) by genotype (G), irrigation treatment (T), year (Y) and their interactions: ns: non-significant; * *p* < 0.05; ** *p* < 0.01; *** *p* < 0.001.

**Table 5:** Mean data (2012–2017) for production and phenolic quality of the six new genotypes and their parentals when grown under sustained deficit irrigation (40–60% ETc).

Genotype	Yield (kg Vine^−1^)	Berry Weight (g)	TPC Skin-Seed (mg kg^−1^ berry)	TPC Quality Group ^¥^	Anthocyanins (mg kg^−1^ berry)	Anthocyanins Quality Group ^¥^
Monastrell	2.83 abc	1.52 d	1528 a	1	939 a	1
Cabernet Sauvignon	3.01 abc	1.06 abc	2220 a	2	1450 a	2
Syrah	3.29 bc	1.49 d	1984 a	1	1583 a	2
MC16	3.33 bc	0.96 ab	3848 b	4	2948 c	4
MC19	3.46 c	1.10 abc	3152 b	3	2713 bc	4
MC72	1.97 ab	0.91 a	3549 b	4	2223 b	3
MC80	1.69 a	1.21 bcd	3970 b	4	2709 bc	4
MS104	3.53 c	1.38 cd	3497 b	4	2913 c	4
MS49	2.11 abc	1.27 bcd	3468 b	4	3191 c	4
**Average**	**2.80**	**1.21**	**3024**	**3**	**2296**	**3**

TPC, total phenol content in skin and seed. Different letters in the same column indicate significant differences among genotypes (Duncan’s multiple range test, *p* < 0.05). ^¥^: classification according to the values shown in Supplementary Table S3.

## Data Availability

All data generated is provided in this manuscript.

## Supplementary Materials

The following supporting information can be downloaded at: https://www.mdpi.com/article/10.3390/plants11101363/s1. Table S1: Meteorological variables recorded in the years 2018, 2019, 2020 and 2021; Table S2: Genetic profile of grapevine material for eight identificatory SSRs. Alleles expressed in base pairs (bp); Table S3: Grape phenolic quality groups based on mean data of six years (2012–2017).
