# Supplementary material for: Productiveness and Berry Quality of New Wine Grape Genotypes Grown under Drought Conditions in a Semi-Arid Wine-Producing Mediterranean Region
Source: Plants (Basel). 2022 May 20;11(10):1363. doi: 10.3390/plants11101363 (PMC9147143; doi:10.3390/plants11101363)
Supplement: Supplementary file 1 [file plants-11-01363-s001.zip › plants-1700136-supplementary.pdf]

plants

MDPI

Supplementary Material

Table S1. Meteorological variables recorded in the years 2018, 2019, 2020 and 2021.

Phenological periods	Period year	ET_O(mm)	Rainfall (mm)	VPD (Kpa)	T_{MAX} (°C)	T_{MED} (°C)	T_{MIN} (°C)	RAD_{AC} (MJ/M^2)	RAD_{MAX} (w/m^2)	RAD_{MED} (w/m^2)
**Year 2018**										
Dormancy	11 Dec-6 April	228.60	107.20	0.54	15.59	8.18	1.19	1421.93	750.03	143.04
Budbreak-Flowering	7 April-22 May	186.04	55.50	0.87	21.62	14.62	6.84	1013.87	1126.48	259.43
Flowering-Veraison	23 May-29 Jul	382.68	41.30	1.69	30.25	22.58	13.29	1774.66	1067.37	307.23
Veraison-Harvest	30 Jul-11 Aug	73.52	0.40	2.20	34.85	26.52	16.63	312.51	975.62	282.93
Harvest-Post harvest	12 Aug-2 Oct	195.45	62.10	1.16	29.58	22.39	15.55	948.03	938.40	214.61
Post harvest-Dormancy	3 Oct-12 Dec	104.77	122.00	0.49	19.17	11.61	5.37	736.61	658.37	122.12
**Total / Annual average**		**1171.06**	**388.50**	**1.16**	**25.18**	**17.65**	**9.81**	**6207.61**	**919.38**	**221.56**
**Year 2019**										
Dormancy	13 Dec-14 April	243.64	25.50	0.57	16.94	7.90	-0.27	1680.96	754.51	160.87
Budbreak-Flowering	15 April-29 May	190.63	118.20	0.92	23.04	15.92	8.18	998.64	1045.22	261.28
Flowering-Veraison	30 May-1 Aug	374.62	2.30	1.89	31.64	23.30	13.02	1723.27	1021.03	317.14
Veraison-Harvest	2 Aug -27 Aug	132.43	22.70	1.84	33.21	25.13	16.13	603.37	984.58	273.43
Harvest-Post harvest	28 Aug- 19 Sep	79.11	147.30	1.00	27.67	21.39	15.33	375.51	910.37	192.28
Post harvest-Dormancy	20 Sep-12 Dec	150.47	118.00	0.63	20.66	13.43	6.58	1026.07	682.05	143.81
**Total / Annual average**		**1170.90**	**434.00**	**1.14**	**25.53**	**17.84**	**9.83**	**6407.82**	**899.62**	**224.80**
**Year 2020**										
Dormancy	13 Dec - 7 April	187.83	204.50	0.44	16.73	8.96	2.19	1364.04	687.76	137.22
Budbreak-Flowering	8 April - 9 May	143.29	28.90	0.74	21.94	15.27	8.24	807.27	1078.32	226.25
Flowering-Veraison	20 May -29 Jul	385.76	9.60	1.73	31.25	22.90	12.79	1884.89	1041.63	312.73
Veraison-Harvest	30 Jul - 17 Aug	99.68	0.40	2.16	35.13	25.83	15.68	461.02	976.63	285.97
Harvest-Post harvest	18 Aug - 14 Sep	122.49	0.00	1.60	31.41	22.67	12.58	609.04	943.93	256.22
Post harvest-Dormancy	15 Sep - 8 Dec	161.61	149.30	0.73	21.85	13.56	5.76	1101.24	703.23	152.51
**Total / Annual average**		**1100.66**	**392.70**	**1.23**	**26.38**	**18.20**	**9.54**	**6227.50**	**905.25**	**228.48**
**Year 2021**										
Dormancy	9 Dec - 10 April	207.94	95.10	0.47	16.04	8.42	1.27	1459.27	707.75	139.65
Budbreak-Flowering	11 April - 22 May	158.91	71.20	0.83	22.51	15.45	7.97	863.33	1079.39	242.00
Flowering-Veraison	23 May - 26 Jul	330.03	96.70	1.52	30.08	22.14	12.79	1625.64	1030.51	294.55
Veraison-Harvest	27 Jul - 25 Aug	142.84	0.20	1.88	33.87	25.50	16.50	658.04	957.20	258.56
Harvest-Post harvest	26 Aug - 22 Sep	96.55	10.90	1.17	29.34	21.83	14.53	478.48	963.68	201.32
Post harvest-Dormancy	23 Sep - 27 Nov	121.39	46.50	0.67	21.04	13.24	5.96	803.27	707.32	143.29
**Total / Annual average**		**1057.66**	**320.60**	**1.09**	**25.48**	**17.76**	**9.84**	**5888.03**	**907.64**	**213.23**

TPC, total phenol content in skin and seed. Different letters in the same column indicate significant differences between genotypes (Duncan's multiple range test, $p<0.05$). ¥: classification according to the values shown in Supplementary Table 3.

Table S2. Supplementary Table 1. Meteorological variables recorded in the years 2018, 2019, 2020 and 2021.

Grapevine material	Vmc1a12	Vmc1e11	Vmc5e9	Vmc8g6	Vviv67	Vvmd5	Vvmd28	Vvmd27
Monastrell	119 137	188 194	214 227	139 173	357 364	223 237	242 256	177 187
Cabernet S.	121 150	192 196	195 218	161 165	364 372	229 237	232 234	173 187
Syrah	137 150	196 206	218 222	169 173	361 381	223 229	216 226	187 189
MC16	119 150	192 194	218 227	139 165	357 372	229 237	234 256	187 187
MC19	137 150	188 196	214 218	161 173	364 364	237 237	232 242	177 187
MC72	121 137	194 196	195 227	139 165	364 372	223 229	234 256	173 177
MC80	121 137	194 196	195 214	165 173	357 364	223 237	232 256	187 187
MS49	137 150	188 206	214 218	173 173	357 381	229 237	226 242	187 187
MS104	119 150	194 196	218 227	169 173	361 364	223 223	226 256	187 187

The genetic profile has made it possible to verify that the evaluated material really comes from the crosses of 'Monastrell' with 'Cabernet Sauvignon' (MC) and 'Syrah' (MS), also confirming that they are 6 new and different genotypes.

Table S3. Grape phenolic quality groups based on mean data of six years (2012-2017).

	TPC skin-seed (mg kg^{-1} berry)			Anthocyanins (mg kg^{-1} berry)		
**Quality group**	Mean	Min.	Max.	Mean	Min.	Max.
1	1836	1550	2121	985	820	1150
2	2218	1960	2476	1381	1127	1635
3	3083	2942	3224	2410	2253	2567
4	3465	3199	3731	2805	2592	3018

TPC, total phenol content in skin and seed. Mean, minimum (Min.) and maximum (Max.) values for each quality group.
